# AMIGO2 attenuates innate cisplatin sensitivity by suppression of GSDME‐conferred pyroptosis in non‐small cell lung cancer

**DOI:** 10.1111/jcmm.17827

**Published:** 2023-07-12

**Authors:** Lian‐kuai Chen, Shu‐ping Lin, Yong‐huan Xie, Xiang‐peng Tan, Ben‐han Xiong, Xiang‐feng Zeng, Cai‐rong Zhu, Shao‐yi Cao, Xiao‐yan Ye, Hong‐jiao Liu, Xiao‐ping Wu

**Affiliations:** ^1^ Institute of Tissue Transplantation and Immunology, College of Life Science and Technology Jinan University Guangzhou China; ^2^ Guangzhou Women and Children's Medical Center Guangzhou Medical University Guangzhou China; ^3^ MOE Key Laboratory of Tumor Molecular Biology Jinan University Guangzhou China; ^4^ Guangdong Provincial Key Laboratory of Bioengineering Medicine Jinan University Guangzhou China

**Keywords:** AMIGO2, GSDME, innate cisplatin sensitivity, non‐small cell lung cancer, pyroptosis

## Abstract

Non‐small cell lung cancer (NSCLC) accounts for approximately 85% of lung cancer. Cisplatin is commonly used in the treatment of many malignant tumours including NSCLC. The innate drug sensitivity greatly affects the clinical efficacy of cisplatin‐based chemotherapy. As a plasma membrane adhesion molecule, amphoterin‐induced gene and ORF‐2 (AMIGO2) initially identified as a neurite outgrowth factor has been recently found to play a crucial role in cancer occurrence and progression. However, it is still unclear whether AMIGO2 is involved in innate cisplatin sensitivity. In the present study, we provided the in vitro and in vivo evidences indicating that the alteration of AMIGO2 expression triggered changes of innate cisplatin sensitivity as well as cisplatin‐induced pyroptosis in NSCLC. Further results revealed that AMIGO2 might inhibit cisplatin‐induced activation of (caspase‐8 and caspase‐9)/caspase‐3 via stimulating PDK1/Akt (T308) signalling axis, resulting in suppression of GSDME cleavage and the subsequent cell pyroptosis, thereby decreasing the sensitivity of NSCLC cells to cisplatin treatment. The results provided a new insight that AMIGO2 regulated the innate cisplatin sensitivity of NSCLC through GSDME‐mediated pyroptosis.

## 1 INTRODUCTION

Lung cancer is the leading cause of cancer‐related mortality worldwide, accounting for nearly one fifth of cancer mortality.^1^ Approximately 85% of the newly diagnosed lung cancer cases belong to non‐small cell lung cancer (NSCLC), and response poorly to most of the therapeutic options.^2^ The cytotoxic chemotherapy agents (e.g. cisplatin) are the first‐line drugs for NSCLC treatment.^3^ Although cisplatin‐based chemotherapy improves the overall survival of NSCLC patients in clinic, many patients are insensitive to the chemical agents and display poor therapeutic efficacy.^4^ Despite intense efforts to explore the regulation mechanisms for the response of tumour cells to chemotherapy, effective targets remain deficient for improving the innate cisplatin sensitivity in NSCLC.

Pyroptosis is a severer type of programmed cell death, which is mediated by gasdermin family members.^5^, ^6^, ^7^ Cells undergoing pyroptosis display distinct characteristics such as ruptured cell membrane with large bubbles blowing from the membrane, increased propidium iodide (PI) uptake and elevated lactic dehydrogenase (LDH) activity.^8^ Recent studies have found caspase‐3 activated by chemotherapy agents can cleave one member of gasdermin family, gasdermin E (GSDME), to generate the N‐terminal fragment (GSDME‐NT), which perforates the plasma membrane and results in pyroptosis.^9^ Moreover, it has been proved that GSDME is associated with clinical cisplatin therapeutic effects, with shorter survival time and higher mortality rate observed in NSCLC patients with lower GSDME expression after treatment with cisplatin.^10^ These results implied that GSDME‐mediated pyroptosis may play an essential role in the innate cisplatin sensitivity in NSCLC.

AMIGO2 is a leucine‐rich repeat (LRR)‐containing cell adhesion molecule initially identified as a neurite outgrowth factor.^11^, ^12^ Further investigations suggest that AMIGO2 is implicated in neuronal survival and regulation,^13^ vascular development and angiogenesis,^14^, ^15^ as well as ischemia protection and immune regulation.^16^, ^17^ Recently, AMIGO2 has been found to play a potential etiologic role in carcinogenesis and tumour progression. SiRNA‐mediated knockdown of AMIGO2 expression in gastric cancer cells resulted in abrogated tumorigenicity.^18^ Forced expression of AMIGO2 promoted tumour cell adhesion to liver endothelial cells and enhanced liver metastasis.^19^ AMIGO2 upregulated in melanomas served as a target gene of bromine domain and extraterminal domain (BET) for melanoma cell survival and metastasis.^20^ Although accumulating evidences suggest that AMIGO2 is a potential target for cancer therapy, the roles and the underlying mechanisms of AMIGO2 in regulation of the chemosensitivity have not been determined. In this study, we proved that AMIGO2 attenuated the response of NSCLC to cisplatin treatment and revealed inhibition of the caspase cascade and GSDME‐mediated pyroptosis served as a novel molecular mechanism for AMIGO2‐triggered drug insensitivity.

## 2 MATERIALS AND METHODS

### 2.1 Cell lines and cell culture

A549 cell line was purchased from National Collection of Authenticated Cell Cultures, while H460 cell line was a kind gift from Professor Liang Chen (MOE Key Laboratory of Tumor Molecular Biology, Jinan University). Cells were cultured in RPMI‐1640 medium supplemented with 10% fetal bovine serum (FBS) and incubated at 37°C in a humidified atmosphere of 5% CO_2_.

### 2.2 Reagents and antibodies

Cisplatin (DDP) was obtained from Yunnan Phytopharmaceutical Co. Ltd. Specific inhibitors of Akt (MK‐2206), PDK1 (BX795), caspase‐3 (Ac‐DEVD‐CHO), caspase‐8 (Z‐IETD‐FMK) and caspase‐9 (Z‐LEHD‐FMK) were purchased from Selleck Chemicals and dissolved in DMSO (MP Biomedicals). The antibodies against cleaved caspase‐3 (cat. no. 9664), cleaved caspase‐8 (cat. no. 9496), cleaved caspase‐9 (cat. no. 7237), Akt (cat. no. 9272), p‐Akt (S473) (cat. no. 4060), p‐Akt (T308) (cat. no.4056), PDK1 (cat. no. 13037), p‐PDK1 (cat. no. 3438), Ki67 (cat. no. 9027), CD34 (cat. no. 3569), GAPDH (cat. no. 2118), anti‐mouse IgG antibody (cat. no. 7076) and anti‐rabbit IgG antibody (cat. no. 7074) were products of Cell Signaling Technology. Anti‐DFNA5/GSDME (cat. no. ab215191) and anti‐AMIGO2 (cat. no. 821607) antibodies were, respectively, obtained from Abcam and ZENBIO.

### 2.3 Generation of stable AMIGO2 knockdown and overexpression cell lines

The recombinant lentiviral plasmid harbouring AMIGO2 shRNA (GCAAGGAAAGTACATTTGTTTCTCGAGAAACAAATGTACTTTCCTTGCTTTTTT) obtained from Hanyang biological company, and the recombinant plasmid pCDH510B/AMIGO2 containing full‐length AMIGO2 coding sequence constructed previously by our lab were cotransfected with packaging auxiliary vectors (psPAX2 and pMD2. G) into 293T cells, respectively, to package lentiviruses, which were applied to infect cells to obtain cell lines stably knocking down or overexpressing AMIGO2 by puromycin screening.

### 2.4 Cell viability assay

Cell viability was measured using the 3‐(4,5‐Dimethyl‐thiazol‐2‐yl)‐2,5‐diphenyltetrazolium bromide (MTT) assay. Cells were seeded in 96‐well plates at 5 × 10^3^ cells/well and cultured overnight. Cells were treated with indicated concentrations of cisplatin for 48 h, or treated with the caspase inhibitor for 4 h prior to administration of cisplatin. 20 μL of MTT (5 mg/mL) was added to each well and the plate was further incubated for 4 h at 37°C. After removal of the culture medium, DMSO (100 μL) was added to each well to dissolve the dark blue crystal. The amount of MTT formazan product was analysed by microplate reader (Model 680; Bio‐Rad) at a wavelength of 570 nm. The half maximal inhibitory concentration (IC_50_) was calculated according to the cell viability values with Prism 6.0 software (GraphPad). Each individual experiment was repeated at least three times.

### 2.5 Flow cytometric analysis

Cells were seeded in 6‐well plates at 3 × 10^5^ cells/well and cultured overnight. Cells were treated with indicated concentrations of cisplatin for 48 h before subjection to analysis of the programmed cell death using the Annexin V‐FITC/PI kit (Life) according to the manufacturer's instructions. Briefly, cells were double stained with Annexin V‐FITC and PI in 1 × binding buffer for 15 min in dark at room temperature, and subjected to flow cytometric analysis using Attune NxT Acoustic Focusing Cytometer (Thermo Fisher Scientific). Data were further analysed with Flowjo v10 software.

### 2.6 Hoechst 33342/PI staining

Cells were seeded in 12‐well plates and cultured overnight. After treatment with indicated concentrations of cisplatin, cells were stained with Hoechst 33342 (5 μg/mL) and PI (2 μg/mL) for 10 min at room temperature, and subjected to observation using Zeiss Axio Observer D1 fluorescence microscope with 20× objective lens (Carl Zeiss Micro‐Imaging GmbH). Five fields per well were randomly selected for counting the stained cells by ImageJ software.

### 2.7 LDH release assay

For assessment of LDH release, cells were seeded in 96‐well plate at 1 × 10^4^ cells/well and cultured overnight. After treatment with cisplatin for 24 h, cell culture supernatants were collected for detection of the LDH activity using LDH Cytotoxicity Assay Kit (Beyotime, Shanghai, China) according to the manufacturer's instructions. Briefly, Aliquots of supernatants were transferred into a blank 96‐well plate at 120 μL/well, and 60 μL of LDH detection solution was added to each well. The plates were then incubated for 30 min in dark at room temperature with gentle shaking. The absorbance was measured at 490 nm on a spectrophotometric microplate reader. The percentage of LDH release was calculated using the equation (LDH_sample_ − LDH_background_)/(LDH_maximum_ − LDH_background_) × 100%, where LDH_sample_, LDH_background_ and LDH_maximum_ are the OD_490_ measured for the drug treated, untreated and lysis solution‐treated supernatants, respectively.

For determination of the LDH activity in tumour tissue using Lactate dehydrogenase assay kit (Nanjing Jiancheng), tumour tissues were cut into pieces and subjected to homogenization in 0.9% normal saline with tissue crusher. The supernatants were collected for measurement of the LDH activity as described above immediately.

### 2.8 siRNA transfection

For silencing of target genes with small interfering RNA, the siRNA (5′‐GUACAAAAGAUCUCGCUGA‐3′) duplexes targeting *AMIGO2* and the siRNA (5′‐GGUCCUAUUUGAUGAUGAA‐3′) duplexes targeting *GSDME* were synthesized by GenePharma, and transfected into cells using Lipofectamine®3000 transfection reagent (Invitrogen) according to the manufacturer's protocols. After grown in a serum‐free medium for 6 h, cells were cultured in RPMI‐1640 medium containing 10% FBS. The knockdown efficiency was determined by real‐time quantitative PCR and western blot analysis.

### 2.9 Real‐time quantitative PCR analysis

Total RNA was extracted using TRIzol™ according to the manufacturer's protocol. Total RNA (1 μg) was reverse transcribed into cDNA using the PrimeScript™ RT reagent Kit (Takara), real‐time quantitative PCR was performed using TB Green® Premix Ex Taq™ (Takara). The sequences of the PCR primers were as follow: AMIGO2: 5′‐CAGAACGGGAAAGTCAGGCT‐3′ (F), 5′‐GACGCCACAAAAGGTGTGTC‐3′ (R); β‐actin: 5′‐GTGAAGGTGACAGCAGTCGGTT‐3′ (F), 5′‐GAAGTGGGGTGGCTTTTAGGAT‐3′ (R). The mRNA level of the detected protein was expressed as the relative ratio to the β‐actin mRNA level. β‐actin was used as an internal control. The relative changes in gene expression were calculated using the 2^−ΔΔ^ *C*q method.

### 2.10 Western blot analysis

Cells or tumour tissues were washed with cold PBS twice and subjected to preparation of protein samples using RIPA lysis buffer (Beyotime). After quantification with BCA protein assay kit (Thermo Fisher Scientific), protein extracts were loaded in 12% SDS‐PAGE gel and transferred onto polyvinyl difluoride (PVDF) membranes (Millipore). The membrane was blocked with 5% nonfat dry milk for 1 h at room temperature, and incubated with primary antibodies at 4°C overnight. After washing three times with TBST (0.1% Tween‐20), the membrane was incubated with anti‐rabbit or anti‐mouse IgG antibodies for 1 h at room temperature. The immunoblots on the membrane was revealed by a BeyoECL Plus kit (Beyotime), and developed on an x‐ray film. Densitometry analysis for western blotting was performed using Quantity One version 4.6.2 software (Bio‐Rad).

### 2.11 Tumour xenograft models

Four‐to‐five‐week‐old BALB/c male nude mice were obtained from GemPharmatech LLC, and maintained under sterile specific pathogen‐free conditions at the Laboratory Animal Center of Jinan University. A549/shNC cells and A549/shAMIGO2 cells (3 × 10^6^) mixed with Matrigel (BD Biosciences) suspended in 100 μL cold PBS were subcutaneously injected to the right flank of the mice. Once the tumour volume reached an average of 100 mm^3^, the mice were randomized into four groups (*n* = 4 in each group). Cisplatin was intraperitoneally injected at a dose of 3 mg/kg every 3 days, whereas the control group received PBS. Body weight and tumour volume of the mice were measured once every 3 days. Tumour volume was obtained using a sliding calliper and calculated as length × width^2^ × 0.52. Tumours were extracted from the sacrificed mice, weighted, imaged and subjected to western blotting and immunohistochemical analysis. The animal experimental procedures complied with the guidelines for animal care of Jinan University (Guangzhou, China), and were approved by the Animal Ethics Committee of this institution.

### 2.12 Immunohistochemical analysis

The tumours were diced, fixed in 4% paraformaldehyde, dehydrated and embedded in paraffin. After deparaffinized, tissue sections were incubated with anti‐Ki67 and anti‐CD34 monoclonal antibodies, followed by secondary antibodies, and indicated by HRP‐streptavidin. Images were acquired using an Inverted Fluorescence Microscope (Zeiss), and subjected to analysis of integrated optical density (IOD) and Area by Image‐Pro® Plus v 6.0.

### 2.13 Statistical analysis

Statistical analysis was performed with GraphPad Prism software. In all experiments, comparisons between two groups were based on two‐sided Student's *t*‐test and one‐way anova was used to test for differences among more groups. *P* values less than 0.05 were considered statistically significant.

## 3 RESULTS

### 3.1 AMIGO2 reduced innate cisplatin sensitivity in NSCLC cells

In order to explore whether AMIGO2 is involved in the innate cisplatin sensitivity of NSCLC cells, stable clones of AMIGO2 knockdown cells A549/shAMIGO2, as well as AMIGO2 overexpressing cells H460/AMIGO2 were constructed and characterized by RT‐qPCR and immunoblotting (Figure S1). The influences of alteration of AMIGO2 expression on innate cisplatin sensitivity were determined by the MTT method. The results indicated that knockdown of AMIGO2 increased the sensitivity to cisplatin treatment, with lower IC_50_ value of A549/shAMIGO2 cells than that of the corresponding A549/shNC cells infected with the control lentivirus. Meanwhile, AMIGO2 siRNA targeting treatment on A549 cells also led to enhanced sensitivity to the cisplatin (Table 1). In contrast, overexpression of AMIGO2 decreased the sensitivity evidenced by the higher IC_50_ value detected in H460/AMIGO2 compared with H460/pCDH control cells (Table 1). The results confirmed that AMIGO2 was implicated in the modulation of innate cisplatin sensitivity in NSCLC cells.

**TABLE 1:** AMIGO2 modulated innate cisplatin sensitivity in NSCLC cells.

Cells	IC_50_/DDP (μg/mL)
A549/shNC	2.3815 ± 0.0145
A549/shAMIGO2	1.6070 ± 0.0530
A549/siNC	3.021 ± 0.2610
A549/siAMIGO2	2.220 ± 0.2630
H460/pCDH	0.8111 ± 0.1239
H460/AMIGO2	1.575 ± 0.4650

### 3.2 AMIGO2 suppressed pyroptosis induced by cisplatin

**FIGURE 1:**
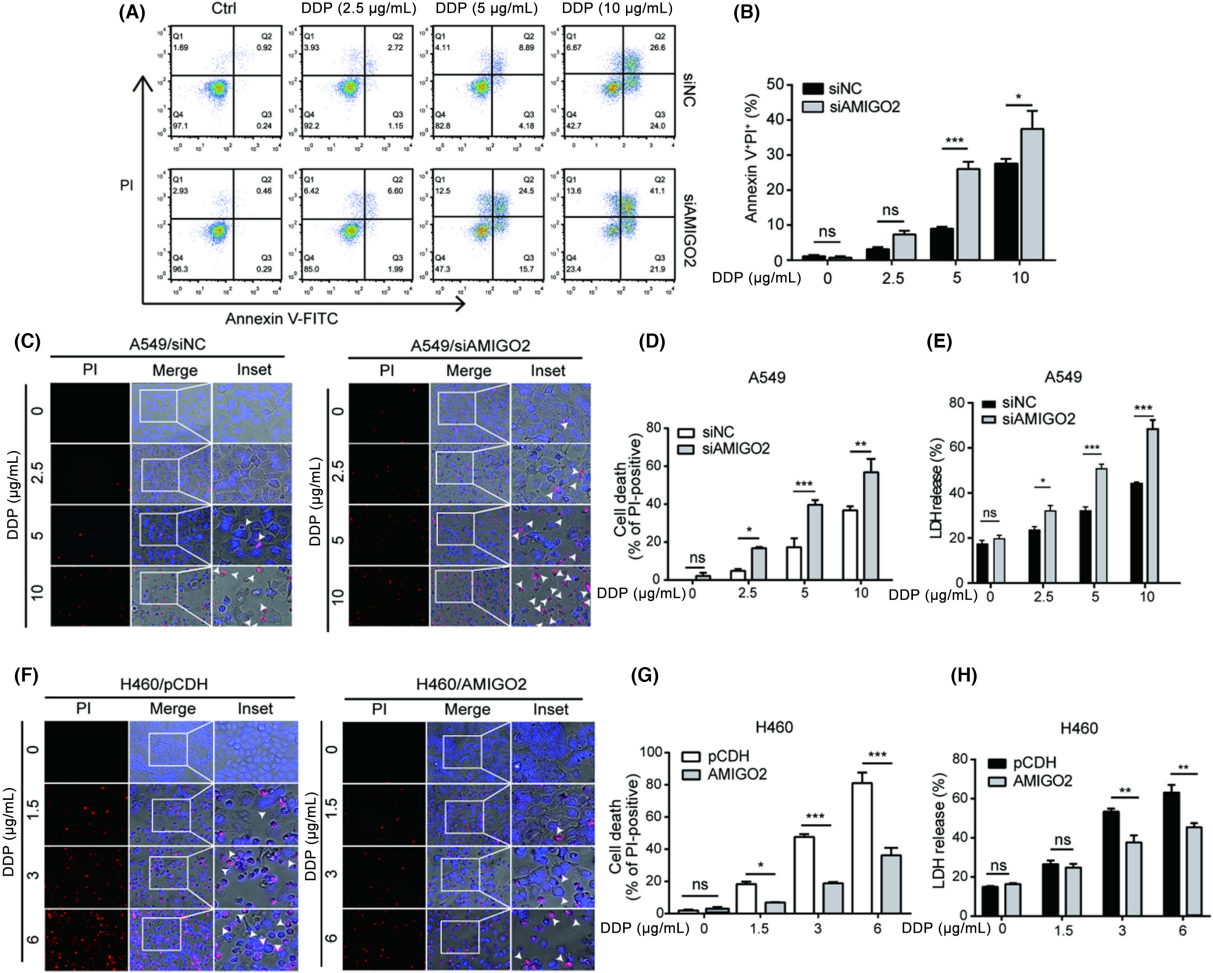
AMIGO2 suppressed pyroptosis induced by cisplatin. (A) A549 cells transfected with AMIGO2 siRNA were treated with graded concentrations of cisplatin (DDP) for 48 h, and subjected to flow cytometric analysis after dual staining with Annexin V‐FITC and PI. Numbers in the representative graph indicated the percentage of cells in each quadrant. Annexin V^+^/PI^+^ displayed the lytic death cells. (B) Quantitative analysis of the ratios of Annexin V^+^/PI^+^ cells. (C) AMIGO2‐silenced A549 cells treated with indicated concentrations of cisplatin for 24 h were stained with 2 μg/mL PI (red) and 5 μg/mL Hoechst 33342 (blue) for 10 min in dark, and observed under an inverted fluorescence microscopy (20× objective lens). White arrow heads indicate PI‐positive cells with large bubbles emerging from the plasma membrane. (D) AMIGO2‐silenced A549 cells with PI‐positive staining were calculated in five random fields prior to statistical analysis. (E) The percentage of LDH release in the culture supernatants from AMIGO2‐silenced A549 cells was measured after treatment with indicated concentrations of cisplatin for 24 h. (F) H460/AMIGO2 cells were treated with cisplatin for 24 h, dual stained with PI (red) and Hoechst 33342 (blue) for 10 min in dark, and then observed by fluorescence microscopy (20× objective lens). White arrow heads indicate PI‐positive cells with large bubbles emerging from the plasma membrane. (G) H460/AMIGO2 cells with PI‐positive staining were calculated in five random fields prior to statistical analysis. (H) The percentage of LDH release in the culture supernatants from H460/AMIGO2 cells was detected after cisplatin treatment for 24 h. *p < 0.05; **p < 0.01; ***p < 0.001; ns, not significant.

Pyroptosis is a type of lytic programmed cell death. Accumulating evidences have suggested that the programmed cell death is associated with the sensitivity of tumour cells to chemotherapy. To further clarify whether pyroptosis played a role in the molecular mechanisms underlying AMIGO2 affecting the response of NSCLC cells to cisplatin treatment, annexin V‐FITC/PI dual staining combined with flow cytometry were at first applied to detect the effects of AMIGO2 knockdown on cell death in A549 cells. The results showed that cisplatin treatment induced lytic cell death (annexin V^+^/PI^+^) in dose dependent manner (Figure 1A). Higher levels of lytic cell death were observed in A549/siAMIGO2 cells compared with A549/siNC control cells (Figure 1B). The results suggested that AMIGO2 suppressed the effects of cisplatin on lytic cell death.

To clarify the features of lytic cell death stained with the annexin V/PI, fluorescence microscopy was further used to examine the morphology of the cells stained with PI, which could penetrate into the lytic cells with impaired membrane integrity. Further results demonstrated that cells treated with cisplatin became round and shrunk with cell membrane integrity being impaired as evidenced by PI‐positive staining (Figure 1C). Interestingly, a typical characteristic of pyroptotic morphology, large bubbles emerging from the plasma membrane, was observed in PI‐positive cells (Figure 1C, white arrow heads). Statistical analysis showed that cisplatin increased the percentage of PI‐positive cells with pyroptotic cell morphology in a dose‐dependent manner in A549/siNC control cells, while knockdown of AMIGO2 strengthened the increased effects of cisplatin on the percentage of PI‐positive cells, accompanying with more AMIGO2‐silenced cells displayed large bubbles compared to the negative control cells (Figure 1C,D). LDH release assay applied for detection of the plasma membrane permeability change further indicated that A549/siAMIGO2 cells exposed to cisplatin released more LDH than A549/siNC control cells (Figure 1E). Similar results were achieved using AMIGO2 knockdown stable cell line A549/shAMIGO2 (Figure S2). Consistent with these observations, we also found that overexpression of AMIGO2 significantly reduced both the percentage of PI‐positive cells with pyroptotic morphology and the LDH release rate in cisplatin‐treated H460 cells (Figure 1F–H). The results implied that AMIGO2 had the potential of alleviating the pyroptosis triggered by cisplatin.

### 3.3 Effects of AMIGO2 on the cisplatin‐activated caspase cascade

**FIGURE 2:**
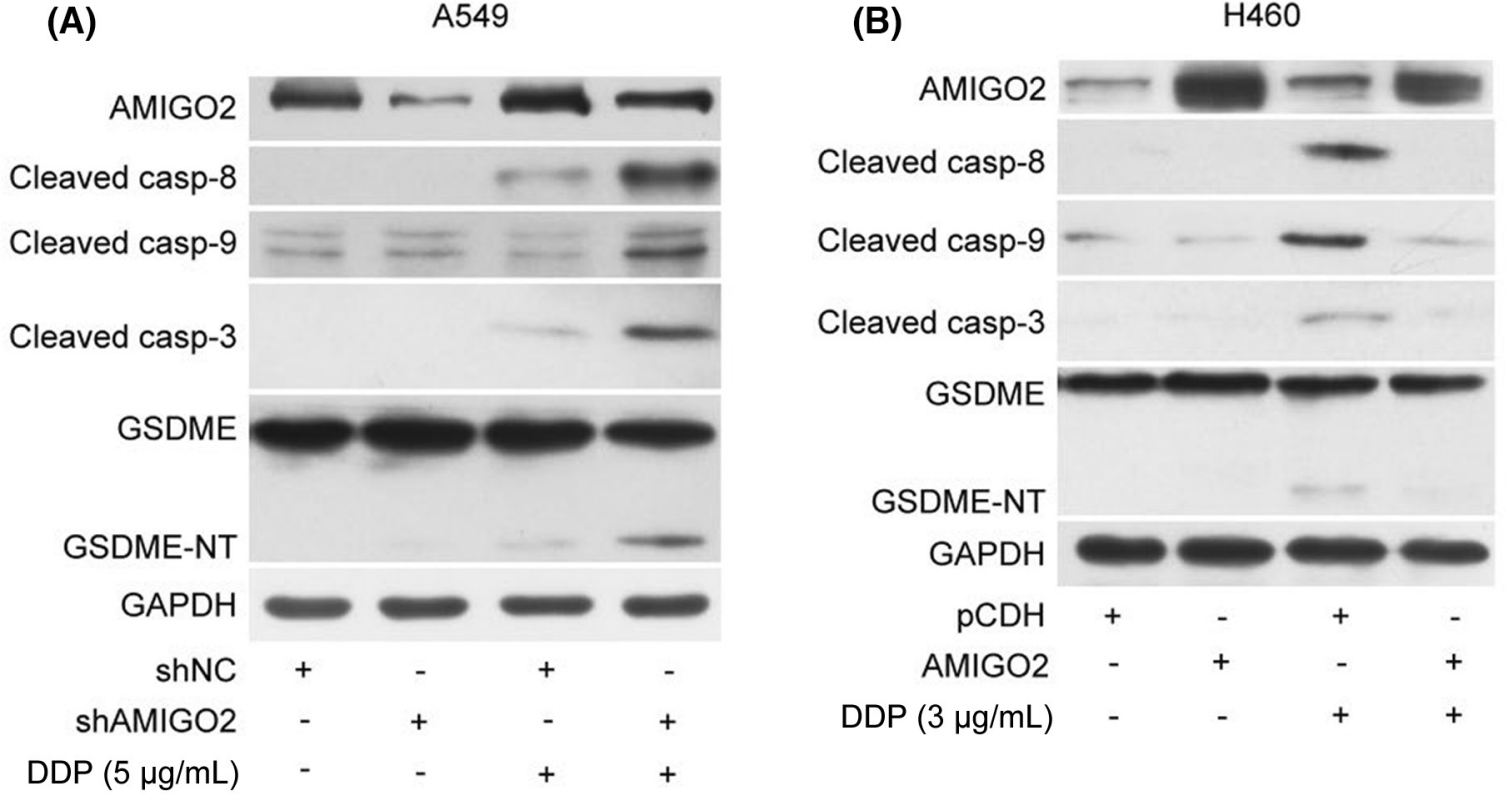
Effects of AMIGO2 on the cisplatin‐activated caspase cascade. (A) A549/shAMIGO2 and the corresponding control A549/shNC cells were treated with 5 μg/mL cisplatin for 24 h, and lysed by 1× SDS‐PAGE loading buffer to obtain protein samples for western blot analysis with the indicated antibodies. (B) H460/AMIGO2 and the corresponding control H460/pCDH cells were treated with 3 μg/mL cisplatin for 24 h before preparation of the protein samples for western blot analysis with the indicated antibodies. GAPDH was used as a loading control.

It has been demonstrated that activation of the caspase cascade can elicit pyroptosis via the proteolytic cleavage of the gasdemin protein family members. To explore the underlying mechanisms by which AMIGO2 inhibited cisplatin‐induced pyroptosis in NSCLC cells, we analysed activations of the caspase cascade by western blotting. As shown in Figure 2, cisplatin significantly activated both extrinsic and intrinsic apoptotic initiators, caspase‐8 and caspase‐9, as well as the downstream executioner caspase‐3. In the cisplatin‐treated cells, we also found that GSDME, a member of the gasdemin protein family recently recognized as a substrate of caspase‐3, was cleaved to generate the GSDME‐NT fragment, which has been proved to be an effector of cell pyroptosis. Importantly, upon cisplatin treatment, AMIGO2 knockdown enhanced activations of the caspase cascade as well as GSDME cleavage in A549 cells (Figure 2A), while enforced expression of AMIGO2 attenuated the caspase and GSDME fragmentation in H460 cells (Figure 2B). The results suggested that AMIGO2 might regulate cisplatin‐induced pyroptosis through the caspase cascade.

### 3.4 AMIGO2 reduced innate cisplatin sensitivity via suppression of GSDME‐mediated pyroptosis

**FIGURE 3:**
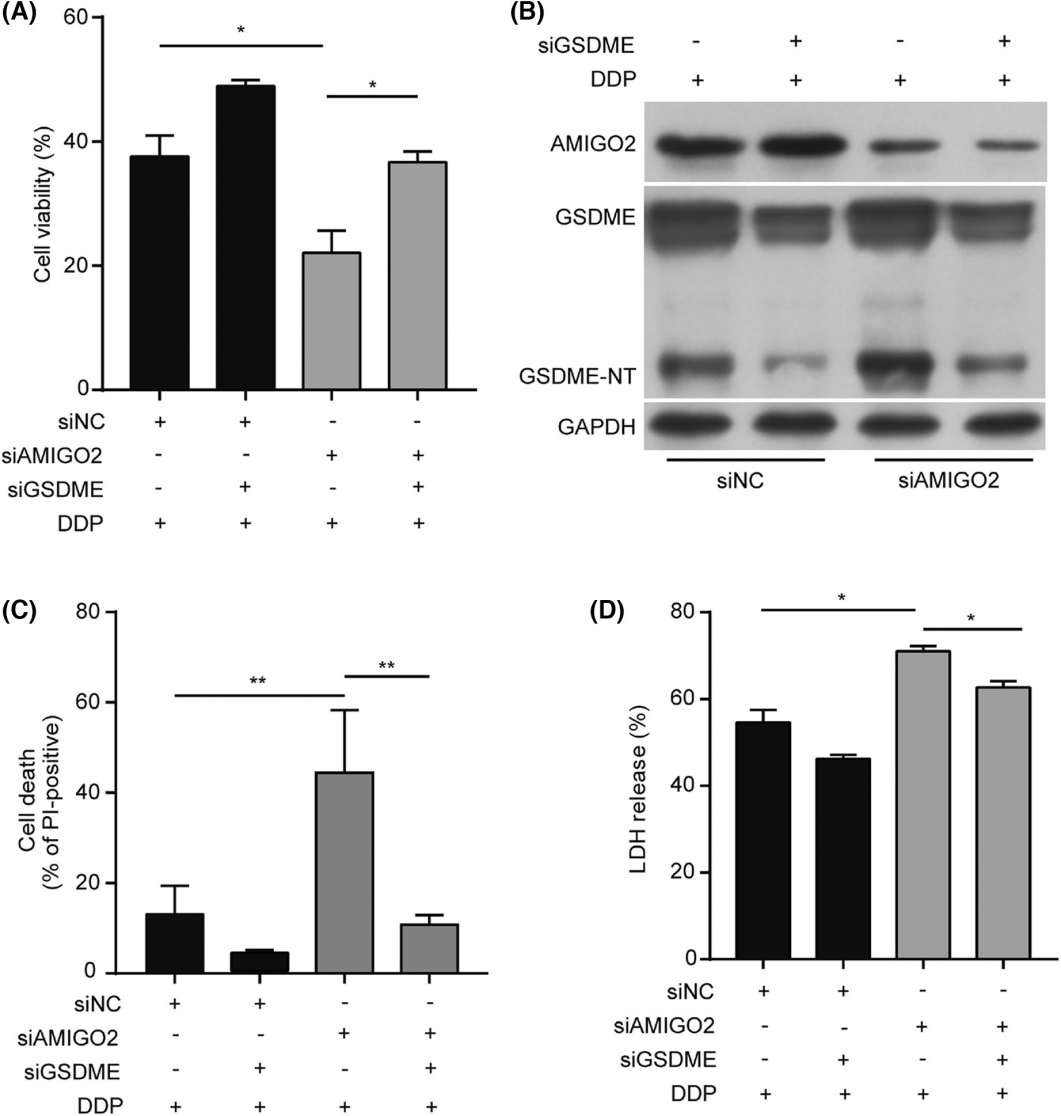
AMIGO2 reduced innate cisplatin sensitivity via suppression of GSDME‐mediated pyroptosis. (A) A549 cells simultaneously transfected with AMIGO2 siRNA and GSDME siRNA were treated with 5 μg/mL cisplatin for 48 h prior to detection of cell viability by MTT method. (B) A549 cells simultaneously transfected with AMIGO2 siRNA and GSDME siRNA were treated with 5 μg/mL cisplatin for 24 h, and subjected to western blot analysis with the indicated antibodies. (C) A549 cells simultaneously transfected with AMIGO2 siRNA and GSDME siRNA were treated with 5 μg/mL cisplatin for 24 h, and dual stained with 2 μg/mL PI and 5 μg/mL Hoechst 33342 for 10 min in dark. After capturing the images by fluorescence microscopy (20× objective lens), PI‐positive cells were calculated in five random fields and statistically analysed. (D) The percentage of LDH release in the culture supernatants from cells treated with cisplatin for 24 h was measured using LDH cytotoxicity assay kit. *p < 0.05; **p < 0.01.

Previous studies have shown that patients with lower GSDME expression levels in tumour tissues have a shorter survival time and higher mortality after cisplatin‐based treatment, implying that the expression levels of GSDME in tumour cells may affect the sensitivity to cisplatin‐based therapy.^10^ To further determine whether GSDME was involved in mediating the effects of AMIGO2 on cisplatin sensitivity, MTT assay was first applied to detect cell viability in A549 cells simultaneously transfected with AMIGO2 siRNA and GSDME siRNA. The results showed that knockdown of GSDME attenuated the suppression effect of AMIGO2 silence on cell survival (Figure 3A). Further investigation indicated that knockdown of GSDME alleviated the promotion effects triggered by AMIGO2 knockdown on cisplatin‐induced pyroptosis as evidenced by less GSDME cleavage, decrease of PI‐positive cells as well as reduced release of LDH were observed in siAMIGO2 and siGSDME‐transfected A549 cells compared with the control cells transfected with siNC (Figure 3B–D). The results suggested that GSDME was involved in AMIGO2 regulating the pyroptosis induced by cisplatin. AMIGO2 might impair the response of NSCLC cells to cisplatin through inhibition of GSDME‐mediated pyroptosis.

### 3.5 AMIGO2 inhibited GSDME‐mediated pyroptosis through (caspase‐8 and caspase‐9)/caspase‐3 cascade

**FIGURE 4:**
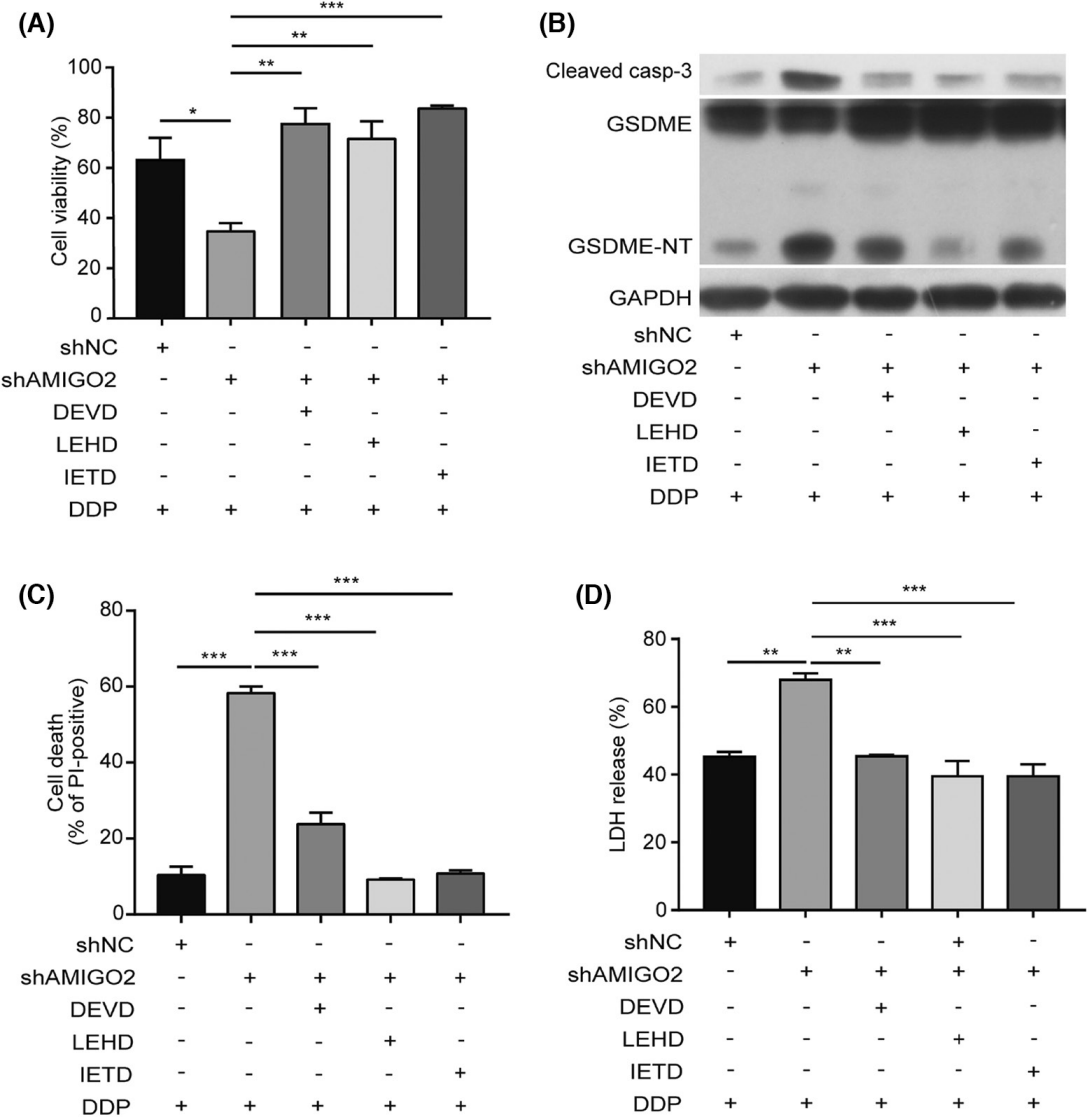
AMIGO2 inhibited GSDME‐mediated pyroptosis through (caspase‐8 and caspase‐9)/caspase‐3 cascade. A549/shAMIGO2 cells pretreated with Av‐DEVD‐CHO (100 μM), Z‐LEHD‐FMK (50 μM) or Z‐IETD‐FMK (50 μM) for 4 h were exposed to 5 μg/mL cisplatin for 24 h. (A) MTT method was performed to assess cell viability. (B) Cells were lysed to obtain protein samples for western blot analysis with the indicated antibodies. (C) Cells were stained with PI and Hoechst 33342 prior to capturing the fluorescent images (20× objective lens). Five fields were randomly selected to calculate PI‐positive cells for statistical analysis. (D) The LDH release in the culture supernatants from cells was detected using LDH cytotoxicity assay kit. *p < 0.05; **p < 0.01; ***p < 0.001.

It has recently been recognized that specific cleavage of GSDME by active caspase‐3 triggers pyroptosis. Combined with the above results indicating that AMIGO2 suppressed the (caspase‐8 and caspase‐9)/caspase‐3 cascade in A549 cells exposed to cisplatin, we speculated that AMIGO2 might inhibit GSDME‐mediated pyroptosis through suppressing the caspase cascade. As expected, pretreatment of the cells with the specific inhibitors of caspase‐8 (Z‐IETD‐FMK), caspase‐9 (Z‐LEHD‐FMK) and caspase‐3 (Av‐DEVD‐CHO) individually attenuated the inhibitory effects of AMIGO2 silence on cell survival (Figure 4A). Western blotting also showed that blockade of caspase‐8, caspase‐9 or caspase‐3 resulted in less GSDME‐NT fragment generated in AMIGO2 knockdown cells (Figure 4B). Consistent with this observation, pharmacological inhibition of caspase activation decreased both the percentage of PI‐positive cells with pyroptotic appearance and the release of LDH in A549/shAMIGO2 cells stimulated with cisplatin (Figure 4C,D). The results implied that AMIGO2 suppressed GSDME‐conferred pyroptosis via a blockade of (caspase‐8 and caspase‐9)/caspase‐3 cascade.

### 3.6 AMIGO2 inhibited pyroptosis through activation of PDK1/Akt (T308) signalling pathway

**FIGURE 5:**
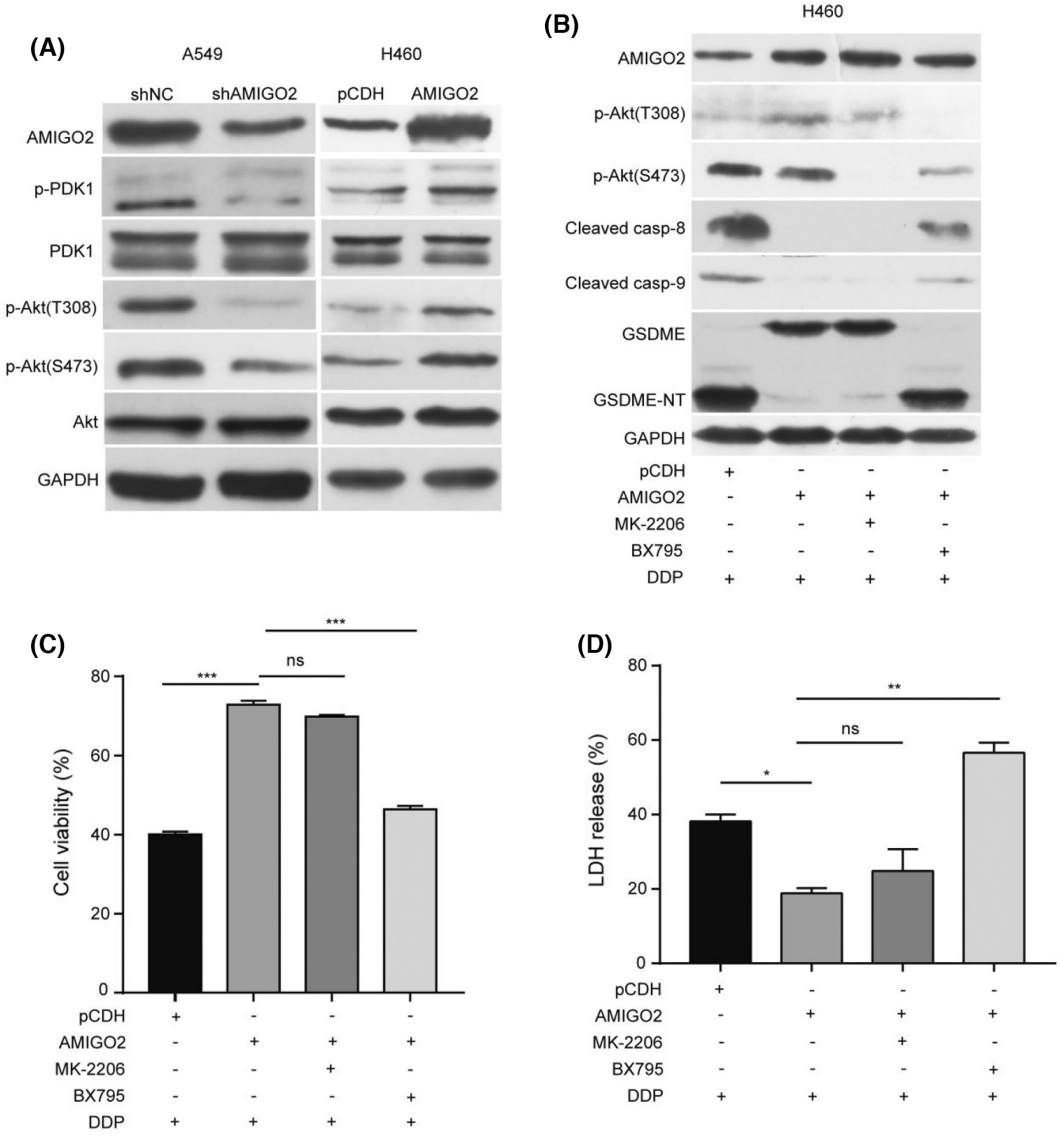
AMIGO2 inhibited pyroptosis through activation of PDK1/Akt (T308) signalling pathway. (A) Proteins extracted from A549/shAMIGO2 and H460/AMIGO2 cells were subjected to western blot analysis with the indicated antibodies. (B–D) H460/AMIGO2 cells pretreated with BX795 (5 μM) or MK‐2206 (1 μM) for 4 h were stimulated with 3 μg/mL cisplatin for 24 h. (B) Proteins in cell lysates were analysed by western blotting with the indicated antibodies. (C) MTT method was performed to assess cell viability. (D) The LDH release in the culture supernatants from cells was assessed using LDH cytotoxicity assay kit. *p < 0.05; **p < 0.01; ***p < 0.001; ns, not significant.

The 3‐phosphoinositide‐dependent kinase 1 (PDK1)/protein kinase B (Akt) signalling pathway plays critical roles in multiple biological processes including cell survival and death. Recent report demonstrated that AMIGO2 is an essential regulator of the PDK1/Akt pathway in endothelial cells (ECs) and modulates the angiogenic function. To determine the relevance of PDK1/Akt signalling pathway in AMIGO2 regulating pyroptosis in NSCLC cells, we first examined the influence of AMIGO2 alteration on the activation of PDK1/Akt pathway. The results showed that either PDK1 or Akt phosphorylation (T308 and S473) was decreased in AMIGO2 deficient cells, whereas overexpression of AMIGO2 enhanced both PDK1 and Akt activation (Figure 5A). It has been known that Akt is phosphorylated at T308 by PDK1 and at S473 by PDK2 family members. We found that the depletion of Akt (T308) activation with BX795 (5 μM), a specific inhibitor for PDK1, promoted the cleavage of both caspase and GSDME, whereas the blockade of Akt (S473) activation with MK‐2206 (1 μM) had no such effect in AMIGO2 overexpression cells (Figure 5B). Concurrent with the above effects on the cleavage of caspase and GSDME, suppression of phosphorylation at T308 rather than at S473 resulted in less cell survival and more LDH release (Figure 5C,D). The results implied that AMIGO2 might inhibit cisplatin‐induced pyroptosis by activating PDK1/Akt (T308) signalling pathway, and thus reduced the sensitivity of NSCLC cells to cisplatin.

### 3.7 Knockdown of AMIGO2 increased cisplatin sensitivity in vivo

**FIGURE 6:**
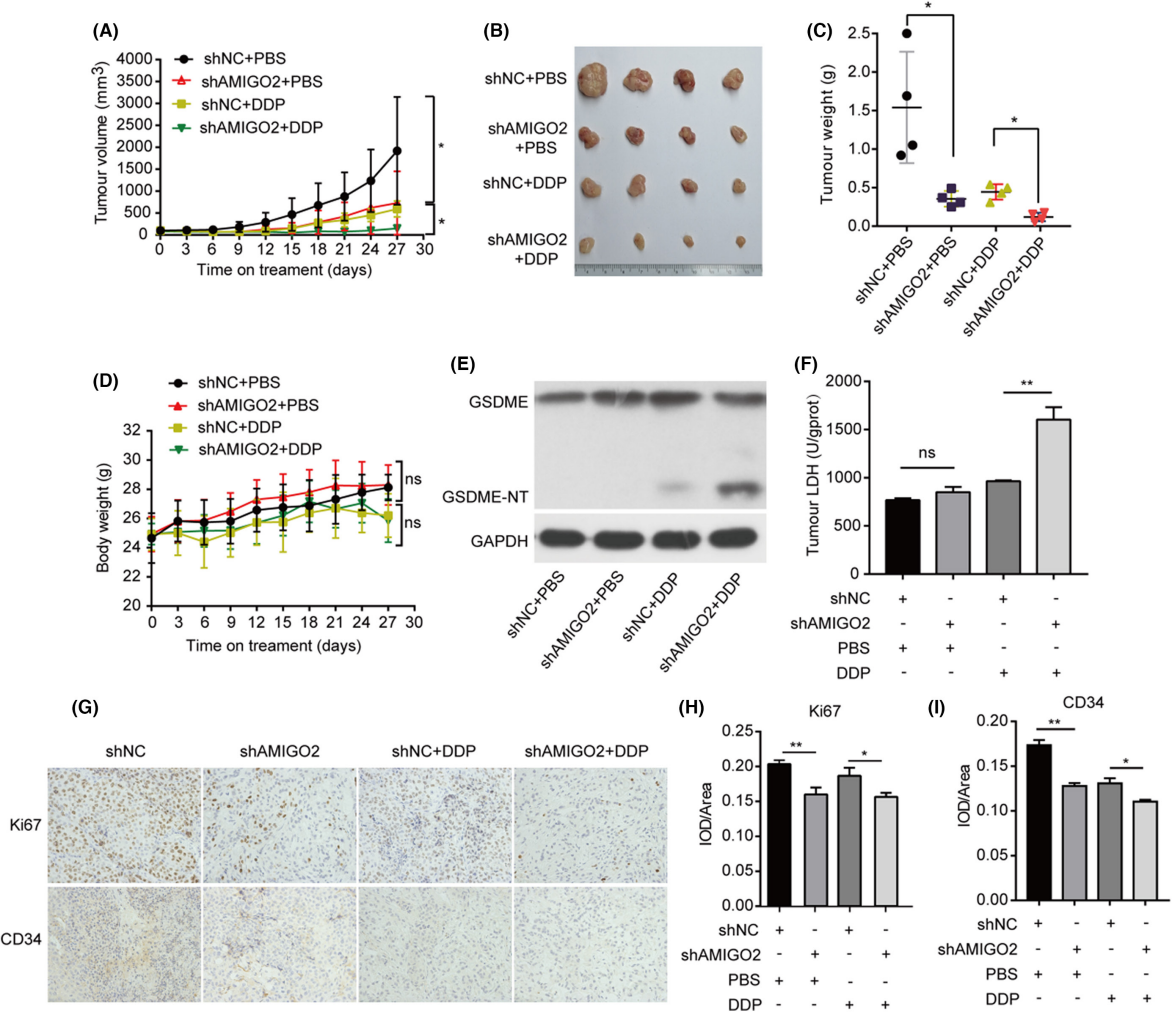
Knockdown of AMIGO2 increased cisplatin sensitivity in vivo. A549/shAMIGO2 or A549/shNC cells (3 × 10^6^) mixed with Matrigel were subcutaneously injected to the right flank of four‐to‐five‐week‐old male BALB/c nude mice. When tumours formed, mice were randomly divided into 4 groups as described in Materials and Methods. The mice received intraperitoneal injections of cisplatin at a dose of 3 mg/kg every 3 days, whereas the control group received PBS. (A) Tumour growth curves were determined by calculation of the tumour volume every 3 days. (B) Photographs of the tumours extirpated from the engrafted mice. (C) Comparison of tumour weights of the indicated groups. (D) Body weights were measured once every 3 days. (E) Tumours were lysed and subjected to western blot analysis of the cleavage of GSDME. GAPDH was used as the loading control. (F) The supernatants from tumour tissue lysate were collected for measurement of the LDH activity using Lactate dehydrogenase assay kit according to the manufacturer's instructions. (G) Tumour sections were immunostained with anti‐Ki67 and anti‐CD34 antibodies. (H, I) Image‐Pro® Plus v 6.0 (for Windows) was used to calculate the integrated optical density (IOD) per unit (Area) of the images. *p < 0.05; **p < 0.01; ns, not significant.

The in vitro study suggested that AMIGO2 possessed the potential of reducing innate cisplatin sensitivity through modulation of GSDME‐conferred pyroptosis in NSCLC cells. In order to explore the effects of AMIGO2 on chemosensitivity in vivo, A549 cells stably transfected with AMIGO2‐specific shRNA (shAMIGO2) or the control shRNA (shNC) were first, respectively, injected into the nude mice to establish the xenograft models. When tumours reached round 100 mm^3^, the cisplatin or PBS (control group) was administrated by intraperitoneal injections once every 3 days to both control shRNA and AMIGO2‐silienced A549 NSCLC tumour‐bearing mice. Compared with control shRNA tumours, AMIGO2‐silienced tumours displayed reduced tumour volume and tumour weight. Intriguingly, AMIGO2 depletion made the tumour more sensitive to the cisplatin treatment as evidenced by a dramatic reduction in both tumour volume and tumour weight in AMIGO2‐silenced tumours than in shNC tumours that received the same dose of cisplatin (Figure 6A–C). Moreover, knockdown of AMIGO2 in tumours caused little effect on the body weight of mice administrated with either cisplatin or PBS (Figure 6D). The results implied that AMIGO2 might be important for tumour growth and responsiveness to cisplatin.

Further western blot analysis of the tumour samples showed that AMIGO2 depletion enhanced the cleavage of GSDME, and LDH levels in mice received the cisplatin treatment (Figure 6E,F). In addition, immunohistochemical assay indicated that knockdown of AMIGO2 significantly suppressed tumour growth and angiogenesis as demonstrated by less Ki67 and CD34‐positive cells in AMIGO2‐silienced tumours compared to the shNC tumours (Figure 6G–I). Similar results were also observed when cisplatin was administrated (Figure 6G–I). Taken together, these findings suggested AMIGO2 depletion enhanced the cytotoxic efficacy of cisplatin through GSDME‐mediated pyroptosis in vivo, which were conformed to the molecular mechanisms as revealed in vitro.

## 4 DISCUSSION

Non‐small cell lung cancer (NSCLC) is the most common type of lung cancers with the highest morbidity and mortality worldwide. Cisplatin is an effective first‐line platinum agent widely used in NSCLC therapy, which has improved the overall survival at 1 year from 5% in untreated patients to 15% in patients administrated with the cisplatin‐based therapy.^21^ However, drug insensitivity commonly observed in clinic greatly affects the cisplatin efficacy, and eventually causes the therapeutic failure. Thus, identification of novel target molecules responsible for modulation of the cisplatin sensitivity and clarification of the underlying mechanisms are essential to design feasible regimen for improving the efficacy of cisplatin in NSCLC treatment.

AMIGO2 is an adhesion transmembrane molecule possessing an extracellular portion with six LRRs and an IgG‐like domain, a transmembrane domain, and a cytosolic region.^11^, ^22^ AMIGO2 has initially been identified as a prosurvival factor of cerebellar granule neurons upon depolarization.^12^ Further evidences suggested AMIGO2 was also implicated in tumorigenesis and tumour progress. Rabenau et al reported that inhibition of AMIGO2 expression led to increased ploidy, chromosomal instability, decreased cell adhesion and abrogation of tumour growth in gastric adenocarcinoma. Interestingly, they also found that significant absolute AMIGO2 expression levels were observed in some lung cancer patients (20%). Of a variety of cancer lines including lung, colon, breast, ovary, cervical, prostate, skin, brain, kidney, liver, osteosarcoma and epidermis, the highest expression observed was in lung A549 and NCI‐H1299 cells, implying that AMIGO2 might play vital roles in a subset of lung cancer.^18^ In this study, AMIGO2 was found to be an essential mediator involved in the response of NSCLC to cisplatin, and depletion of AMIGO2 markedly enhanced innate sensitivity against cisplatin via GSDME‐mediated pyroptosis in NSCLC cells. However, further clinical investigations are needed to elucidate the correlation between AMIGO2 expression and innate cisplatin sensitivity in NSCLC patients.

Pyroptosis is a form of programmed cell death originally thought to be executed by the gasdermin D (GSDMD) subjected to cleavage by the active caspase‐1, ‐4, ‐5 or ‐11 to release its gasdemin‐NT fragment with pore‐forming activity.^23^, ^24^, ^25^ Recent study found that another gasdermin, GSDME, competitively suppressed the association of caspase‐3 with its apoptotic substrates by binding to caspase‐3, and thus switch apoptosis to pyroptosis.^9^ It has been proved that cisplatin could activate caspase‐3 and induce pyroptosis in both cancer and normal cells with abundant GSDME expression.^9^, ^26^ Combined with the literature that indicated that GSDME promoted cisplatin sensitivity to inhibit lung carcinoma by mediating pyroptosis to trigger antitumor immunocyte infiltration,^10^ we speculated that caspase‐3‐related cascade and its substrate GSDME might be implicated in the mechanisms of AMIGO2 affecting innate sensitivity against cisplatin. We first demonstrated that AMIGO2 suppressed the caspase and GSDME fragmentation induced by cisplatin. Knockdown of GSDME or blockade of caspase activations with the specific inhibitors further revealed that AMIGO2 impaired the cisplatin sensibility through suppression of the cisplatin‐induced activation of (caspase‐8 and caspase‐9)/caspase‐3 cascade and the subsequent GSDME‐conferred pyroptosis.

The Akt signalling pathway is associated with multiple physiological and pathological processes, including cell survival, angiogenesis, as well as cancer and neurological disorders.^27^, ^28^ The activation of Akt is triggered by phosphorylation at two sites, T308 and S473. PDK1 served as a primary activator of Akt is autophosphorylated at the site of S241 via binding to phosphatidylinositol‐3,4,5‐triphosphate (PIP3), resulting in the recruitment of PDK1 to the membrane and the subsequent phosphorylation of Akt at one site T308.^29^ Another site at S473 was phosphorylated by PDK2 family members such as mTORC2 (mammalian target of rapamycin complex 2) or DNA‐PK (DNA‐dependent protein kinase).^30^, ^31^ Recent report indicated that AMIGO2 served as a scaffold protein at the plasma membrane to promote PDK1 membrane localization and PDK1/Akt activation in endothelial cells (ECs) and regulated the angiogenic function.^14^ Consistent with these suggestions, we found that knockdown of AMIGO2 significantly inhibited the phosphorylation of PDK1 (S241), Akt (T308) and Akt (S473), while enhanced the expression of AMIGO2 stimulated the activations of the above signal molecules in NSCLC cells. Given AMIGO2 can promote the phosphorylation of Akt at both T308 and S473, whose initial phosphorylation was triggered by PDK1 and PDK2 family members respectively, it is reasonable to speculate that in addition to modulation of PDK1 activity for Akt phosphorylation at T308, AMIGO2 might have the potential of regulating the kinase activity of PDK2 family members for Akt phosphorylation at S473. Further investigations are required to clarify the possible roles of AMIGO2 in regulation of the PDK2 family members.

It has been known that the activated Akt not only directly inhibits the protease activity of caspase‐9 by phosphorylating its serine residue at position 196 (S196),^32^ but also impairs the kinase activity of the caspase‐8 activator, ASK1 (apoptosis signal‐regulated kinase 1) via phosphorylating its serine residue at position 83 (S83), and thus indirectly abrogates the protease activity of caspase‐8.^33^, ^34^ Activation of Akt by AMIGO2 might be responsible for inhibition of caspase‐3 by repressing both caspase‐9 and caspase‐8, resulting in suppression of GSDME cleavage and the subsequent cell pyroptosis, thereby reducing the cisplatin sensitivity of NSCLC cells. Intriguingly, we found that inhibition of Akt phosphorylation at S473 with MK‐2206 had little effect on the protease activity of caspase‐9 and caspase‐8, as wells as the GSDME fragmentation and pyroptosis, whereas abrogation of Akt phosphorylation at T308 with BX795 significantly enhanced the cleavage of caspase‐9, caspase‐8 and GSDME, and promoted pyroptosis in cisplatin‐treated NSCLC cells overexpressing AMIGO2. The results suggested that phosphorylation of Akt at T308 rather than at S473 was essentially indispensable for the inhibitory effects of AMIGO2 on the caspase cascade and GSDME‐mediated pyroptosis.

In summary, we have first provided evidences to reveal the role and the underlying mechanism of AMIGO2 in regulation of the innate cisplatin sensitivity in NSCLC cells. Our results demonstrated that AMIGO2 might inhibit cisplatin‐induced activation of (caspase‐8 and caspase‐9)/caspase‐3 via stimulating PDK1/Akt (T308) signalling axis, leading to attenuation of GSDME‐conferred pyroptosis, thereby reducing the innate sensitivity of NSCLC cells against cisplatin (Graphical Abstract). Therefore, determination of the expression levels of AMIGO2 in NSCLC patients might give the hint for predicting the efficacy of cisplatin treatment. More importantly, AMIGO2 might be employed as an adjunctive therapeutic target for improving the response to cisplatin‐based chemotherapy in a subset of NSCLC with abundant GSDME expression.

## AUTHOR CONTRIBUTIONS

**Lian‐kuai Chen:** Investigation (lead); methodology (lead); validation (equal); writing – original draft (lead). **Shu‐ping Lin:** Data curation (lead); methodology (equal); visualization (lead). **Yong‐huan Xie:** Data curation (equal); methodology (equal); validation (lead). **Xiang‐peng Tan:** Investigation (equal); validation (equal). **Ben‐han Xiong:** Formal analysis (equal); visualization (equal). **Xiang‐feng Zeng:** Methodology (equal); validation (equal). **Cai‐rong Zhu:** Resources (lead); validation (equal). **Shao‐yi Cao:** Methodology (equal); resources (equal). **Xiao‐yan Ye:** Methodology (equal). **Hong‐jiao Liu:** Funding acquisition (equal); project administration (lead). **Xiao‐ping Wu:** Conceptualization (lead); funding acquisition (lead); supervision (lead); writing – review and editing (lead).

## CONFLICT OF INTEREST STATEMENT

The authors declare that there are no conflicts of interest.

## Supporting information


Figure S1

## Data Availability

The data that support the findings of this study are available from the corresponding author upon reasonable request.
