# Supplementary material for: AMIGO2 attenuates innate cisplatin sensitivity by suppression of GSDME‐conferred pyroptosis in non‐small cell lung cancer
Source: J Cell Mol Med. 2023 Jul 12;27(16):2412–23. doi: 10.1111/jcmm.17827 (PMC10424296; doi:10.1111/jcmm.17827)
Supplement: Supplementary file 1 — Figure S1 [file JCMM-27-2412-s001.docx]

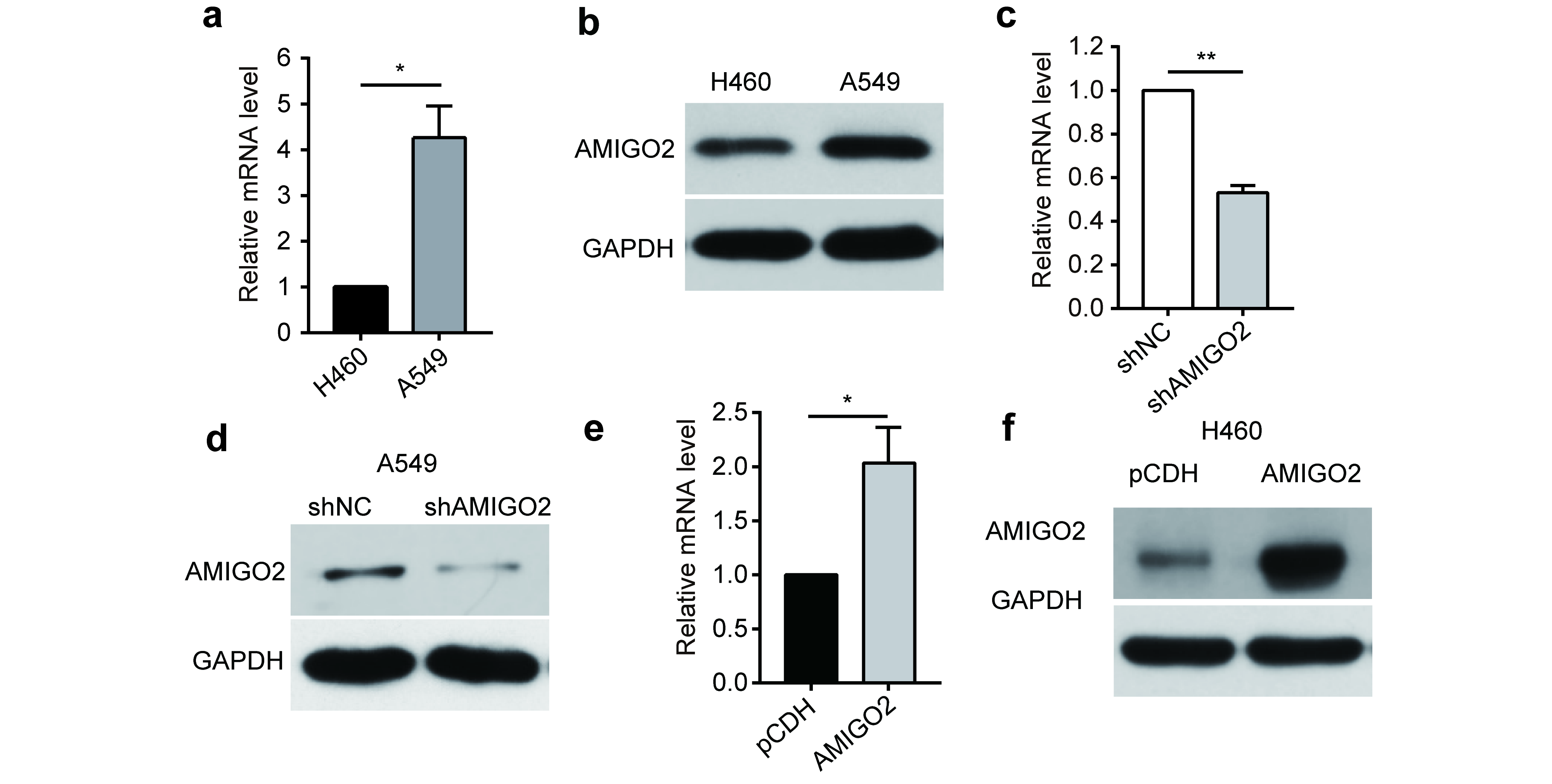


**Fig.S1**


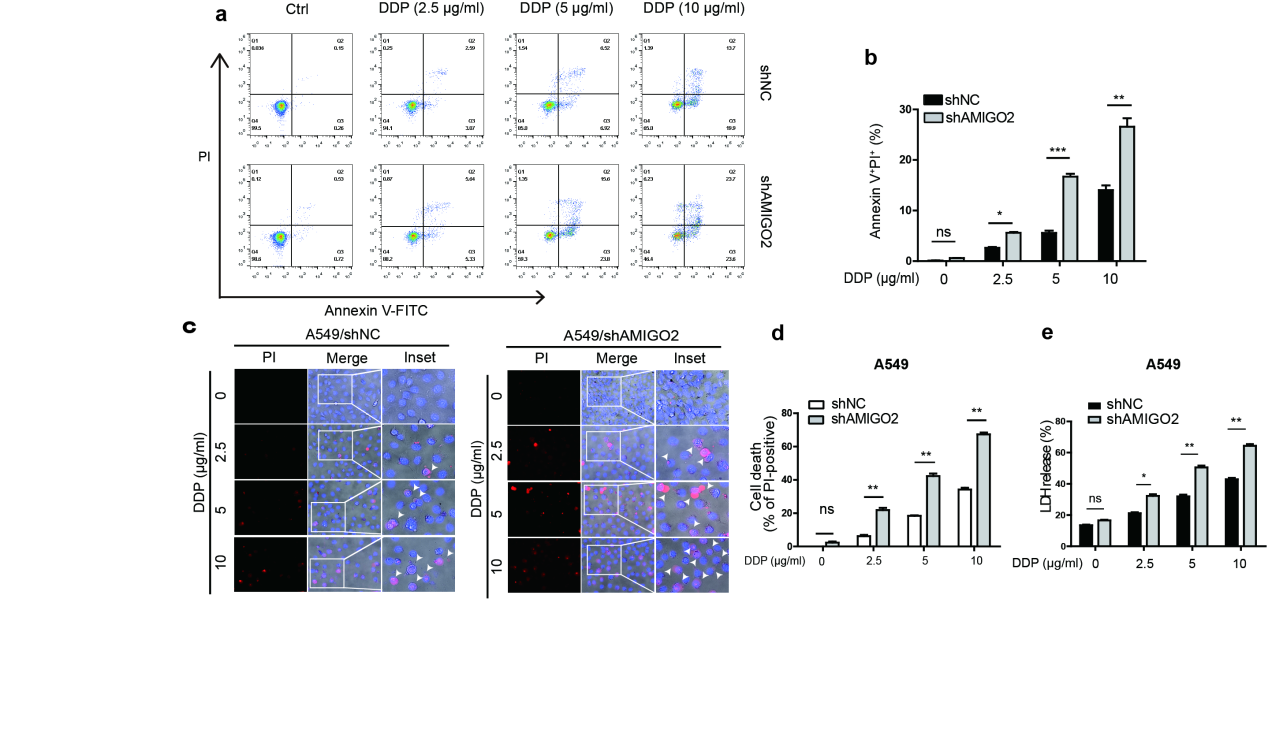


**Fig.S2**

**Fig. S1 Construction of AMIGO2 knockdown cells A549/shAMIGO2 and AMIGO2 overexpressing cells H460/AMIGO2.** (a, b) Comparison of mRNA and protein expression levels of AMIGO2 between A549 and H460 cells. (c, d) Semi-quantitative RT-PCR combined with western blotting were applied to detect the mRNA and protein levels of AMIGO2 in A549/shAMIGO2 and the corresponding control A549/shNC cells. (e, f) Confirmation of AMIGO2 overexpression in H460/AMIGO2 cells obtained by puromycin screening by RT-PCR as well as western blot analysis. * *p* <0.05; ** *p* <0.01.

**Fig. S2 AMIGO2 knockdown by shRNA promoted cisplatin-induced pyroptosis.** (a) A549/shAMIGO2 cells were treated with graded concentrations of cisplatin (DDP) for 48 h, and subjected to flow cytometric analysis after dual staining with Annexin V-FITC and PI. Numbers in the representative graph indicated the percentage of cells in each quadrant. Annexin V^+^/PI^+^ displayed the lytic death cells. (b) Quantitative analysis of the ratios of Annexin V^+^/PI^+^ cells. (c) A549/shAMIGO2 cells treated with indicated concentrations of cisplatin for 24 h were stained with 2 μg/ml PI (red) and 5 μg/ml Hoechst 33342 (blue) for 10 min in dark, and observed under an inverted fluorescence microscopy (20× objective lens). White arrow heads indicate PI-positive cells with large bubbles emerging from the plasma membrane. (d) A549/shAMIGO2 cells with PI-positive staining were calculated in five random fields prior to statistical analysis. (e) The percentage of LDH release in the culture supernatants from A549/shAMIGO2 cells was measured after treatment with indicated concentrations of cisplatin for 24 h. * *p* <0.05; ** *p* <0.01; *** *p* <0.001; ns, not significant.
